# Sustainable antibullying program implementation: School profiles and predictors

**DOI:** 10.1111/sjop.12487

**Published:** 2018-09-17

**Authors:** Miia Sainio, Sanna Herkama, Tiina Turunen, Mikko Rönkkö, Mari Kontio, Elisa Poskiparta, Christina Salmivalli

**Affiliations:** ^1^ University of Turku Finland; ^2^ University of Jyväskylä Finland

**Keywords:** School‐based intervention, antibullying program, sustainable implementation, evidence‐based program, victimization

## Abstract

We examined the sustainability of the KiVa antibullying program in Finland from its nationwide roll‐out in 2009 to 2016. Using latent class analyses, we identified four different patterns of implementation. The *persistent* schools (43%) maintained a high likelihood of participation throughout the study period. The *awakened* (14%) had a decreasing trend during the first years, but then increased the likelihood of program participation. The *tail‐offs* (20%) decreased in the likelihood of participating after the third year, and the *drop‐offs* (23%) already after the first year. The findings suggest that many schools need support during the initial years to launch and maintain the implementation of evidence‐based programs; yet a large proportion of schools manage to sustain the program implementation for several years. The logistic regression analyses showed that large schools persisted more likely than small schools. Lower initial level of victimization was also related to the sustainability of the program. Finally, persistent program participation was predicted by several school‐level actions during the initial years of implementing the program. These results imply that the sustainability of evidence‐based programs could be enhanced by supporting and guiding schools when setting up the program during the initial implementation.

## Introduction

During past years, bullying at schools and its prevention has attracted major attention worldwide (United Nations, 2015). To answer to this demand, a handful of evidence‐based antibullying programs, and some key characteristics that make these programs effective have been identified (Ttofi & Farrington, 2011; Vreeman & Carroll, 2007). However, the development of an effective program is only the first step in the bullying prevention process (Durlak & DuPre, 2008). To have the desired long‐term outcomes for student safety and well‐being, successful programs and practices need to be scaled up and eventually sustained over time (Fixsen, Blase & Fixsen, 2017). Scaling up and sustaining a program in a real‐life setting is, however, quite different from short‐term evaluations or trials often characterized by participation of program developers. To achieve long‐term benefits, it is important to understand program implementation after such trials end, and to identify factors which help sustaining evidence‐based practices over time.

Studies on sustainability of school‐based programs are emerging (e.g., Andreou, McIntosh, Ross & Kahn, 2015; McIntosh, Mercer, Nese & Ghemraoui, 2016; McIntosh, Mercer, Nese, Strickland‐Cohen & Hoselton, 2016; Woodbridge, Sumi, Yu *et al*., 2014), including research on the sustainability of antibullying programs during their initial trials (Ahtola, Haataja, Kärnä, Poskiparta & Salmivalli, 2013; Haataja, Ahtola, Poskiparta & Salmivalli, 2015), and first few years of implementation (Leadbeater, Gladstone & Sukhawathanakul, 2015). Nevertheless, research on long‐term sustainability of antibullying programs is currently lacking. We address this gap by examining the sustainability of KiVa antibullying program in Finland. By using data collected across seven years, we discover different patterns of program implementation as well as school‐level factors that predict persistent program implementation.

## Sustainability of program implementation

Sustainability refers to continued use of a program over time, particularly after the active support associated with initial program implementation ceases (e.g., Scheirer & Dearing, 2011). As straightforward as this definition is, there are no commonly used definitions, procedures, or a research paradigm guiding research on sustainability (Scheirer & Dearing, 2011). As a consequence, previous studies have utilized various operational definitions of sustainability (Moore, Mascarenhas, Bain & Straus, 2017; Scheirer, 2005). Some studies have used retrospective evaluations of continuation, institutionalization, and duration of a project (e.g., Savaya & Spiro, 2012), while others have attempted to take into account the fidelity of implementation by determining criteria for adequate delivery (McIntosh, Mercer, Nese, Strickland‐Cohen, *et al*., 2016). The latter approach is in accordance with the definition if sustainability used by Han and Weiss (2005, p. 666) as “continued implementation of an intervention or prevention program, with ongoing implementation fidelity to core program principles”. Yet, operationalizing fidelity itself is fairly complex given the various aspects through which the concept can manifest (i.e, adherence, dose, quality of delivery, participant responsiveness, and program differentiation, Dane & Schneider, 1998; Dusenbury, Brannigan, Falco & Hansen, 2003). Requirement that fidelity is taken into consideration when assessing sustainability of implementation is further complicated by the fact that programs tend to be modified over time (Scheirer & Dearing, 2011). Indeed, some researchers view adaptations or modifications of programs as natural or even necessary to achieve sustainability (Harn, Parisi & Stoolmiller, 2013; Owens, Lyon, Brandt *et al*., 2014).

Studies of sustainability also differ in the time horizons taken (Moore *et al*., 2017). It is common that program continuation is determined at a specific time point, for instance, after two to five years after the initial implementation period (e.g., Andreou *et al*., 2015; Leadbeater *et al*., 2015; Woodbridge *et al*., 2014), but other approaches have been introduced as well. For example, McIntosh, Mercer, Nese and Ghemraoui (2016) took into account the possible changes in implementation activity over time by tracking annually whether the program was implemented or not. While investigating School‐wide Positive Behavioral Interventions and Supports program, they found two sustained (*sustainers* and *slow starters*) and two non‐sustained trajectories of implementation (*late abandoners* and *rapid abandoners*). Based on the trajectories, they concluded that the first and the third year of implementation are the most likely time points for abandoning the program, whereas it may take three to five years for some schools to achieve an adequate level of implementation. Their study demonstrated that several years of data collection are required to capture the full range of different implementation trajectories.

## Factors that predict sustainability

A number of potential predictors of sustainable program implementation have been identified in empirical studies using either qualitative (Andreou *et al*., 2015; Leadbeater *et al*., 2015; Sanford DeRousie & Bierman, 2012; Woodbridge *et al*., 2014) or quantitative approaches (McIntosh *et al*., 2013; McIntosh, Mercer, Nese & Ghemraoui, 2016; McIntosh, Mercer, Nese, Strickland‐Cohen, *et al*., 2016). Despite that sustainability has been defined and operationalized in somewhat different ways across studies, three broad categories of factors predicting sustainable implementation can be identified: (1) the project itself; (2) the organizational setting; and (3) the broader community (Shediac‐Rizkallah & Bone, 1998; see also Scheirer & Dearing, 2011).

The project‐ or program‐related factors pertain to both how programs are designed and implemented. For instance, a school‐based program needs to fit in the school and classroom environments, be easy to use, and be flexible enough to allow some adaptations (Andreou *et al*., 2015; Sanford DeRousie & Bierman, 2012; Woodbridge *et al*., 2014). On the implementation front, multiple studies have highlighted that support from program developers is positively related to sustainability (Andreou *et al*., 2015; Leadbeater *et al*., 2015; Woodbridge *et al*., 2014). Quite naturally, program effectiveness has also been shown to be important for sustainability (Shediac‐Rizkallah & Bone, 1998); however, perceived effectiveness of the program and its components may be valued more than evidence provided by evaluation trials (Andreou *et al*., 2015; Sanford DeRousie & Bierman, 2012; Woodbridge *et al*., 2014).

Organizational factors enhancing sustainability are also numerous. For instance, McIntosh, Mercer, Nese and Ghemraroui (2016) discovered that elementary schools were more likely to sustain chosen practices than middle schools, and larger schools were more likely to sustain a program than smaller schools. Leadership and administrative support for the program have also been identified as important factors for sustainability (Andreou *et al*., 2015; Haataja *et al*., 2015; Leadbeater *et al*., 2015; Sanford DeRousie & Bierman, 2012; Woodbridge *et al*., 2014). Moreover, staff motivation (buy‐in) and the importance of internal champions for the program have been brought up in several studies (Andreou *et al*., 2015; Leadbeater *et al*., 2015; Woodbridge *et al*., 2014). In contrast, staff turnover has been identified as a challenge for program sustainability, but this may be mitigated by engaging each and every one within the school community (Leadbeater *et al*., 2015), and inviting new staff members to join the program teams (Andreou *et al*., 2015). Overall, creating a unified culture in which a program is embedded in the language and codes of conduct of the school is perceived important for sustainability (Andreou *et al*., 2015; Leadbeater *et al*., 2015). Leadbeater *et al*. (2015) also point out that regular staff meetings are required in creating ongoing communication among staff and renewing of commitments to sustain the program. Also, both individual and organizational level values are often associated with sustainability. If a given program does not correspond to the values of the school, precious time is unlikely to be allocated to program activities (Andreou *et al*., 2015; Leadbeater *et al*., 2015; Sanford DeRousie & Bierman, 2012).

Finally, factors related to the broader community, including socioeconomic and political landscape, can influence whether programs are maintained (Shediac‐Rizkallah & Bone, 1998). For instance, educational policies, such as the flexibility of curriculum, and the written policies prioritizing the program values support program implementation (Andreou *et al*., 2015; Leadbeater *et al*., 2015). Sustainability of a program may also be enhanced when it is adopted by several schools in the district (McIntosh, Mercer, Nese & Ghemraoui, 2016). McIntosh and colleagues offered two possible explanations for this finding. First, it is possible that close proximity to other schools using the program promotes networking and sharing of ideas between the schools, thus enhancing sustainability. Second, it is possible that the finding is due to school district level effects, such as some districts having more resources than others.

In sum, a number of factors could be related to sustainability of evidence‐based programs in educational settings. However, while there are multiple perspectives on the issue, there are also important limitations in current research. By far, most studies are retrospective staff interviews in schools that have implemented a certain program, thus lacking the perspective of non‐sustainers (e.g., Andreou *et al*., 2015; Leadbeater *et al*., 2015; McIntosh *et al*., 2013; Woodbridge *et al*., 2014). The few exceptions utilizing a longitudinal quantitative framework have focused on demographic characteristics of the schools, or community level factors in predicting sustainability (McIntosh, Mercer, Nese & Ghemraoui, 2016; McIntosh, Mercer, Nese, Strickland‐Cohen, *et al*., 2016). These studies have a limited potential to inform practical aspects of program implementation, because the studied predictors of sustainability are largely outside the control of the schools. In order to provide comprehensive school‐level guidelines to maintain evidence‐based practices in the long term, we need to identify predictors of sustainability that schools themselves can influence, such as actions taken during the initial phases of program implementation.

## KiVa antibullying program

The KiVa antibullying program is based on the participant role approach and aims to reduce bullying by influencing bystander responses (Salmivalli, Lagerspetz, Björkqvist, Österman & Kaukiainen, 1996). The program is based on a whole‐school approach and includes both universal actions to prevent bullying and indicated actions to stop ongoing bullying. The universal actions consist of lessons delivered to students, including KiVa lessons targeted to Grades 1 and 4, and KiVa themes to Grade 7. The KiVa lessons/themes are accompanied by three age‐specific online games for the targeted grade levels. Furthermore, antibullying awareness in school is raised by KiVa symbols (posters and recess supervisors' vests). To aid in program implementation, the school staff is provided with materials for a kick‐off event for students and for a staff meeting, and presentation graphics and newsletters for introducing KiVa to the parents. To implement the indicated actions to address acute bullying cases, a KiVa team is formed from school staff. Finally, KiVa schools monitor their progress in antibullying work and bullying levels with feedback based on annual student and staff online surveys (see for more about the program, Salmivalli, Kärnä & Poskiparta, 2010).

As explained earlier, the political and legislative landscape can influence both the uptake and sustainability of programs. In Finland, the changes to the Basic Education Act in 1998 and 2003 (FINLEX, 1998) were important steps towards more systematic national level antibullying work. A more concrete approach was taken in 2006, when the Ministry of Education and Culture funded the University of Turku research team to develop an antibullying program for basic education. The program was tested in a randomized controlled trial during 2007–2009 with promising results (Kärnä *et al*., 2013; Kärnä, Voeten, Little, Poskiparta, Kaljonen & Salmivalli, 2011). This positive evidence encouraged nationwide dissemination of the program, enabled by the funding from the Ministry of Education and Culture until 2011.

In sum, KiVa was developed to meet the need for concrete tools to address bullying in a favourable political and legislative environment. The program was attractive for schools because it had succeeded in demonstrating effectiveness, was affordable (free of charge), and available (all schools in Finland were invited to register). Scaling up the program was successful, and by the end of 2011, the program had reached 90% of Finnish schools offering basic education (Herkama, Saarento & Salmivalli, 2017). Furthermore, the student survey data from the first year of nationwide roll‐out provided evidence that the program was effective also under real‐world conditions (i.e., with larger sample, and with less support from the program developers than during the initial trial; Kärnä, Voeten, Little, Poskiparta, Alanen & Salmivalli, 2011), and subsequent annual student survey have demonstrated that bullying and victimization continue to decrease in the schools responding the surveys (Herkama *et al*., 2017). However, while the number of participating schools was impressive, the responding activity to the surveys has declined over the years. Because the long‐term impact of the program requires continuing implementation, an important question deserves attention: which factors support schools in sustainable implementation?

## The present study

We examine the sustainability of the KiVa antibullying program in Finland from its nationwide roll‐out in 2009 to 2016 using annual survey data. Our study consists of two parts. In the first part, we identify various patterns of implementation. Instead of relying on retrospective measures, or using activity at an arbitrary time point to indicate whether the program is being sustained or not, we use the schools' responding activity to the annual online student survey across years as an indicator for sustainable implementation. This is in accordance with views that sustainability is not necessarily a steady state (Bumbarger & Perkins, 2008; Scheirer & Dearing, 2011), but instead varies over time. Following the procedure by McIntosh, Mercer, Nese and Ghemraoui (2016), we uncover latent classes of schools with different patterns of activity over time. This design is motivated by the idea that while we expect to find persistent schools, there are likely to be also schools that struggle with implementation – some sooner and others later. This information can provide insight into the critical time points when the schools need more support in order to sustain the programs they adopted.

In the second part of the study, we identify factors predicting the sustainability of the KiVa program in Finland. Our focus is on school‐level factors (Shediac‐Rizkallah & Bone, 1998) that could have practical significance for planning sustainability, that is, factors that can be influenced and emphasized when setting up the implementation in the school. We expect that assigning a person in charge (i.e., having internal champions for the program, Andreou *et al*., 2015; Leadbeater *et al*., 2015; Woodbridge *et al*., 2014), planning implementation carefully (Haataja *et al*., 2015), and raising awareness of the program (Andreou *et al*., 2015; Leadbeater *et al*., 2015) during the initial years predict sustainability. Moreover, the actual use of various program elements during the initial years is expected to be associated with persistent implementation.

The initial levels of victimization may also be related to sustainability. On the one hand, it is possible that schools with more severe bullying problems are more receptive to an antibullying program simply because they see more need for the program. On the other hand, the relationship could also be reverse; a low level of problems can indicate that a school is generally more receptive to antibullying practices and may even implement some already. This prior experience in antibullying work may make it easier for them to implement a new program in a sustained way. Therefore, we control for the initial level of victimization in the analyses, as it might be related to program sustainability in one way or the other.

Finally, following earlier research (McIntosh, Mercer, Nese & Ghemraoui, 2016), we examine demographic factors, namely school type (elementary versus middle school) and school size as predictors. We expect results similar to the earlier study by McIntosh, Mercer, Nese and Ghemraoui (2016); the sustainability of the KiVa program is expected to be more likely in elementary than in middle schools, and larger schools may be more likely to sustain implementation.

## Method

### Sample

The sample consists of Finnish basic education schools registered as KiVa program users during 2009–2016.^1^ In order to have a more homogenous sample, we restricted the sample to the schools starting the implementation of the program in 2009 (*n* = 1,459) or 2010 (*n* = 818). After excluding schools that had been closed or combined with other schools during the years 2009–2016 (*n* = 400), and schools not providing any responses in either the student or the staff survey during the focal years (*n* = 106), our final sample size was 1,771 schools. Of these included schools, 64.3% had started to implement KiVa in 2009 and 35.7% in 2010; 68.8% were elementary schools, 14.2% middle schools, and 17.0% combined schools with both elementary and middle school students. Finnish was the official language in 89.9% of the schools and the rest provided education in Swedish, the other official language in Finland. School size ranged from 13 to 960 students, the average school size being 230 (*SD* = 168) students.

### Measures

The data consists of the responses to the annual online student and staff surveys that take place at the end of each school year between the end of April and the beginning of June. The student survey has been conducted since the spring of 2009. To provide a baseline against which to compare the effects of the program, schools start administering the student survey in the spring prior to the launch of the program implementation (i.e., the baseline was spring 2009 for schools that started KiVa in fall 2009, whereas it was spring 2010 for schools that started in fall 2010).The student survey was expanded on 2011 to include questions measuring the implementation of the KiVa program components, and awareness of the program (i.e., after one or two years of implementing the program, depending on the school's registry year).

The staff survey was administered for the first time after the first year of program implementation (i.e., KiVa Year 1). One person from each school responds to this survey on behalf of the school. This survey has also been expanded over the years, and although the survey has been available since 2010, all the questions used in the present study were added in 2011.

We considered the initial years of implementation to be crucial for integrating the program to the school practices. Therefore, we focused on the implementation characteristics from the years 1 to 3 as predictors of sustainability. To do so, we averaged all predictors over the first three years, except for the school size and type, and the baseline measure for victimization.

#### Program participation (student survey)

Annual monitoring of the level of victimization is a core component of the KiVa program. Therefore, participating in the student survey is a natural index of schools' program participation. Each year, participation was coded as 1 if students from the school responded to the survey and 0 if students did not respond.

#### School size and type

Upon the registration to the KiVa program, schools provided background information on the number of students enrolled and school type (elementary, middle, or combined). To keep this background data up to date, the schools' KiVa contact persons were asked to update the information annually. We used the across years' average for school size, whereas the most recent value was used for school type in the few cases where there were changes. For the analysis predicting persistent participation, we created a binary variable: schools including middle school level, that is, being either middle schools or combined schools with both elementary and middle school grade levels (= 1) versus elementary‐only schools (= 0).

#### Victimization (student survey)

Baseline victimization was measured by one item from Olweus Bully/Victim questionnaire (Olweus, 1996): “How often have you been bullied at school in the last two months?”. Students answered on a five‐point scale (0 = *not at all,* 1 *= once or twice,* 2 *= 2 or 3 times a month,* 3 *= about once a week,* 4 *= several times a week*). These data came from the pre‐implementation survey and the responses were averaged at school level to produce the school‐level measure of baseline victimization.

#### Coordination (staff survey)

Staff members were asked “Has the school appointed a person/persons who is/are familiar with the program and who coordinate/s the implementation of the program as a whole and guide/s others in matters concerning the program” (0 = *No*, 1 = *Ye*s).

#### Planning (staff survey)

Staff members reported whether the school had a written plan on: (1) which grade levels will be targeted by KiVa lessons/themes (despite recommendations, schools themselves made the final decision); (2) when the KiVa team discussions to address the bullying cases are organized (e.g., is there a fixed slot for them in the schedule or some other point in time such as recess, after school, etc.); (3) how bullying cases are directed to the KiVa team; and (4) how to inform parents about cases tackled by the team. Each question was responded by selecting either *Yes* (= 1) or *No* (= 0). The four variables were averaged as one composite measure of implementation planning.

#### Informing (staff survey)

Staff members reported whether students, parents, and staff members were informed about the KiVa program and whether the KiVa survey results were presented to the same parties. The exact questions were: (1) “Has your school organized a staff meeting day/discussion about KiVa for the whole school staff?” (0 = *No*, 1 = *Yes*); (2) “Have the students in the school been informed about KiVa (so that the very least, everyone knows that your school is a KiVa school)” (0 = *No*, 1 = *Yes*); (3) “Have parents/guardians been informed about KiVa (so that the very least, everyone knows that your school is a KiVa school)?” with response options *No*;* Yes*,* a copy of the online Parent's Newsletter has been send to homes*; and *Yes*,* we have organized a Back‐to‐School Night about KiVa*. (0 = *No*, 0.5 = *one positive option*, 1 = *both positive options*); (4–6) “Have the survey results been presented to staff/students/parents?” (each responded (0 = *No* or 1 = *Yes*). All items were averaged.

#### Awareness (student survey)

The students were asked “Is KiVa program used in your school?” (0 = *No/I don't know*, 1 = *Yes*). The responses were aggregated to school‐level by averaging student responses, thus representing the proportion of students being aware of the KiVa program each year.

#### Vests (student survey)

The students were asked “Have the recess supervisors in your school been wearing bright vests with KiVa logo?” (0 = *No*,* not at all*, 1 = *Yes*,* to some extent*, 2 *= Yes*,* all supervisors*). The responses were aggregated to school‐level by averaging.

#### Lessons/themes (student survey)

As the student lessons/themes were targeted only to the grades 1, 4, and 7, only the responses from students in these grade levels (1, 4, and 7) were used. Students were asked “Have you had KiVa lessons/themes delivered in your class since last fall?” (0 = *No*, 1 = *Yes*). The responses were aggregated to school‐level by averaging.

#### Online game (student survey)

The students in the target grade levels (1, 4, and 7) were asked whether they have played online KiVa games since last fall?” (0 = *No*, 1 *= Yes*,* during the lessons or KiVa theme days at school* or *Yes*,* outside of school*. The responses were aggregated to the school‐level by averaging.

### Analyses

The data were analyzed using Mplus 7 (Muthén & Muthén, 1998). First, following McIntosh, Mercer, Nese and Ghemraoui (2016), in order to identify implementation profiles, we used latent class analysis (LCA). LCA reveals mutually exclusive hidden or unobserved groups (latent classes) from the data based on response patterns in multivariate data (see e.g., Oberski, 2016). This approach has the advantage that no particular functional form of time is imposed on the classes (Feldman, Masyn & Conger, 2009). To safeguard against estimates that converge to local instead of a global maximum of the likelihood function, we reran all models with 100 random starts and compared the log likelihoods from the replications to the original log likelihood (Masyn, 2013): 91 of the replications produced the same likelihood value and class frequencies as the main result. To further inspect the possibility of two equal peaks in the likelihood, we rerun the model with five different seeds to verify that they produced the same parameter estimates. The optimal number of classes was determined by fitting the LCA model with increasing number of classes (Asparouhov & Muthén, 2012; Nylund, Asparouhov & Muthén, 2007; Oberski, 2016). Because LCA is an exploratory method, the final solution was determined by considering jointly the statistical indices, model parsimony, and interpretability of the profiles (Oberski, 2016). Second, we used logistic regression analysis to examine which factors predicted membership in the persistent group.

### Missing data

Table 1 shows the number of schools from which students and staff, respectively, responded to the survey across the implementation years. Some of the data are missing by design because the schools that started the program implementation in 2010 do not have data for the KiVa Year 7, and because the schools that started in 2009 had no implementation questions for their Year 1 survey.

**Table 1:** Number of schools (and percentage relative to the number of schools registered during 2009–2010, N = 1,877) responding to the student and staff surveys

	Student survey		Staff survey	
	*n*	%	*n*	%
Baseline	1576	(84.0%)		
KiVa Year 1	1373	(73.1%/)*	267	(14.2%)*
KiVa Year 2	1183	(63.0%)	607	(32.3%)
KiVa Year 3	1048	(55.8%)	532	(28.3%)
KiVa Year 4	1006	(53.6%)	492	(26.2%)
KiVa Year 5	966	(51.5%)	419	(22.3%)
KiVa Year 6	956	(50.9%)	434	(23.1%)

Notes

Schools that registered to the KiVa program in 2010 do not have data for the KiVa Year 7. *Questions on implementation were introduced to both student and staff surveys in 2011; therefore, only schools that started in 2010 could respond these questions during KiVa Year 1 resulting in a significantly lower response rate on Year 1. In student survey the number of students responding to questions on implementation was 530 (28.2%).

In the LCA focusing on the 1,771 schools that provided any data during the seven years, the missing data on student survey in a particular year was an indication of non‐response that year, and accordingly coded as zero, except for the design‐based missing values in Year 7 for the cohort that started in 2010. These values were coded as missing values for the LCA and the model was estimated using full information maximum likelihood estimation (FIML; Enders, 2010) to account for the missing data.

In the logistic regression analyses, the independent variable was derived from the LCA, and had no missing values. The predictors, after averaging across three years, however, had 48.5% missing in the staff responses on coordination, planning, and informing, 20.6% in student responses on awareness and using vests and 18.5% in having had lessons and played the game. The baseline victimization had missing values in 11.0%. Here the missing at random assumption (MAR) made by FIML was possibly violated. In practice, a few of the predictor variables could be missing not at random (MNAR), meaning that whether a value is missing depends on the value itself. This is particularly a problem with the staff survey; it is plausible that low levels of the specific program component implementation predict missing answers to these questions because both depend on the overall degree of commitment to the program by the school. To assess this possibility, we ran another set of analyses using the program participation variables from the student surveys as auxiliary variables. The results were virtually the same as the original FIML results. We further assessed the potential impact of missingness in the staff variables by running the analyses on subsets of the data where the lowest values (i.e., the least active schools) on these variables were eliminated. The results from these subsample analyses were very similar to the main analysis results. Therefore, our data does not indicate that there is a MNAR problem.

While we do not have evidence of MNAR being a problem for our analysis, it is ultimately impossible to test for MNAR empirically. Thus, it is prudent to speculate what the effects of MNAR would be to our results if the data on the staff data were MNAR. Because the staff variables are only weakly correlated with the student and demographic variables (Table 4), it is unlikely that missingness in the staff variables had a great impact on the estimated effects of the student and demographic variables. Because the estimated correlations between the staff variables are low, we can assess the impact of MNAR in these variables through the bivariate relationships between each of the staff variables and program implementation. If the missing data were from schools that were generally less active, then these observations would be less likely to be in the persistent group and also have smaller values on the staff variables. Censoring the data this way will cause the bivariate relationships to become smaller or more negative, which must be kept in mind when interpreting the results.

## Results

### Implementation profiles

In the first part of the study, we identified implementation profiles using latent class analysis. The optimal number of latent classes was determined by estimating the model multiple times, increasing the number of latent classes one at a time and comparing two consecutive sets of model estimates using the Lo‐Mendell‐Rubin test (LMR), bootstrapped likelihood ratio tests (BLRT), and information criterion indices Akaike's information criterion (AIC), Bayesian information criterion (BIC), sample‐ size adjusted BIC (aBIC) produced by Mplus (Asparouhov & Muthén, 2012). These statistics were chosen because they are conventionally used for deciding the number of classes in LCA and were available in Mplus. The information criterion indices have a weakness that they are descriptive indices (non‐inferential) and are not guaranteed to provide a hard stopping rule for adding classes (Masyn, 2013). The simulation study by Nylund *et al*. (2007) indicates that aBIC index has a generally superior performance over the AIC and BIC and is therefore preferable. The two likelihood ratio tests have as an advantage over the information criterion statistics that they are inferential statistics that provide a statistical test for the null hypothesis that k – 1 classes explain the data. Of the two statistics considered here, LMR and BLRT, the latter has been shown to have generally superior performance in both detecting that a k class solution fits better than a k – 1 class solution and not indicating that k classes would be required when the population only has k – 1 classes (Nylund *et al*., 2007).

The fit statistics are presented in Table 2. The information criterion indices changed only a little between the four and five class solutions and the BIC index started increasing when the fifth group was added, indicating that the difference of fit between the four‐ and five‐class solutions was not meaningfully large. The BLRT statistic indicated that each additional class explained the data statistically significantly better than the previous model with one less class. However, given our large sample size and the high power of the BLRT test (Nylund *et al*., 2007), it is possible that these differences, while non‐zero are not meaningfully large. Therefore, we proceeded with a more detailed inspection of the four and five class solutions to see if there were meaningful differences between the two. A cross‐tabulation of the predicted most likely class variables from the four‐ and five‐class solutions and the inspection of the class profiles revealed that the potential five‐class solution basically split the class with decreasing trend from the Year 3 (tail‐offs, see below) to one with fast decreasing and another with slowly decreasing implementation profile. As this five‐class solution was not meaningfully different from the four‐class solution, we chose the more parsimonious four‐group solution.

**Table 2:** Fit indices for the latent class analyses by number of groups

Latent classes	AIC	BIC	aBIC	LMR	BLRT	Entropy
1	16659.11	16702.94	16677.53			
2	13490.85	13584.00	13529.99	*p* < 0.001	*p* < 0.001	0.86
3	13229.96	13372.42	13289.82	*p* = 0.026	*p* < 0.001	0.74
4	12970.08	13161.85	13050.66	*p* < 0.001	*p* < 0.001	0.77
5	12942.65	13183.74	13043.96	*p* = 0.085	*p* < 0.001	0.77
6	12929.59	13219.99	13051.61	*p* = 0.521	*p* < 0.001	0.78

AIC = Akaike's Information Criterion, BIC = Bayesian Information Criterion, aBIC = sample‐size adjusted BIC, LMR = Lo‐Mendell‐Rubin adjusted likelihood ratio test; BLRT = bootstrapped likelihood ratio test.

**Figure 1:**
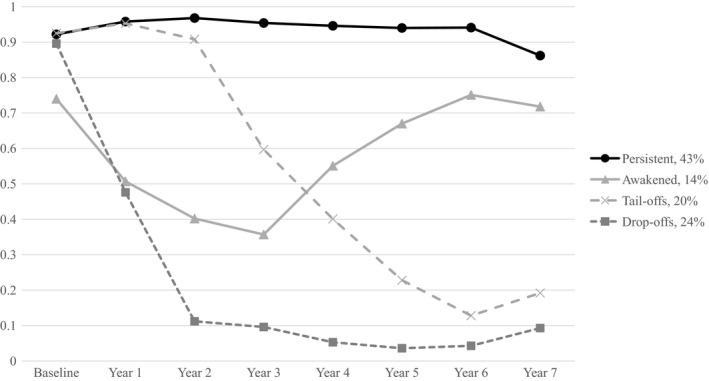
Latent class analyses (LCA) on 1,771 schools that started KiVa program 2009–2010 based on responding to student survey across years.

We analyzed the classes by assigning each case to the most likely class based on the LCA analysis. The four implementation profiles obtained from LCA are shown in Fig. 1. The largest group (*n* = 757; 42.7%) is labeled as *persistents*. The estimated probability of program participation in this group was high every year (range 0.86–0.97). The second largest group was the *drop‐offs* (*n* = 416; 23.5%). These schools had high probability for participation in the baseline survey, but by the second year the estimated probability declined to close to zero. The *tail‐offs* was also a relatively large group (*n* = 358; 20.2%) with a declining trend in participation starting from Year 3. The fourth group, the *awakened* (*n* = 240; 13.6%), started lower than average, showed an initial decline in participation, but then increased in participation.

We examined whether the four groups differed in school size, type and language (Table 3). Persistent schools were larger than other schools and drop‐offs were the smallest. Persistent schools were less often primary schools and more often middle or combined schools, whereas drop‐offs were more often primary schools and less often combined schools than expected by chance. Finally, awakened schools were unlikely to be middle schools.

**Table 3:** Group differences on background variables

	Persistent (42.7%)	Awakened (13.6%)	Tail‐off (20.2%)	Drop‐off (23.5%)	Difference test for metric and categorical variables	
School size, *M* (*SD*)	277^a^ (162)	218^b^ (160)	217^b^ (170)	165^c^ (190)	F(3,1758) = 40.61	*p <* 0.001
Primary	63.5%^a^	74.2%^bc^	67.6%^ab^	76.2%^ac^	χ2(6) = 29.58	*p <* 0.001
Middle	16.4%^a^	9.6%^a^	13.7%^a^	13.5%^a^		
Combined schools	20.1%^a^	16.3%^ab^	18.7%^a^	10.3%^b^		
Finnish speaking	91.5%	91.3%	88.9%	86.9%	χ^2^(3) = 6.80	*p* = 0.079
Start year 2009	61.8%	68.8%	66.5%	64.2%	χ^2^(3) = 4.83	*p* = 0.184

Note

Same superscript means no differences between groups.

### Predicting persistence

To identify factors predicting sustainability of the KiVa program, more specifically, the membership in the persistent group identified by the LCA, we applied logistic regression analyses. Due to the probabilistic nature of the latent class variable, the most likely class variable can be tough to contain measurement error (Asparouhov & Muthén, 2014). However, the estimated sensitivity and specificity of the persistent class were 94.9% and 95.1% in the LCA analysis indicating that the effects of misclassification of this particular class were likely small.^2^ The estimated means, standard deviations, and correlations for all the variables are provided in Table 4. The correlations among predictors are for the most part small or modest. School size and type are correlated (0.46); middle schools tend to be larger than elementary schools. There is less victimization in larger schools (–0.20) and in middle schools, as compared with elementary schools (–0.29). Playing the online game and using the recess supervisors' vests (*r* = –0.26 and –0.36, respectively) is less common in middle schools than in elementary schools.

**Table 4:** FIML estimated means, standard deviations, and correlations of the study variables

		*M*	*SD*	1	2	3	4	5	6	7	8	9	10	11
1	Middle school	0.31	(0.46)	–										
2	School size	229.72	(168.33)	0.46	–									
3	Initial victimization	0.73	(0.24)	–0.29	–0.20	–								
4	Coordination	0.97	(0.14)	–0.02	0.01	0.02	–							
5	Planning	0.81	(0.21)	0.05	0.10	–0.05	0.14	–						
6	Informing	0.65	(0.20)	–0.09	–0.02	–0.03	0.13	0.19	–					
7	Awareness	0.79	(0.11)	–0.11	–0.05	–0.04	–0.04	0.13	0.23	–				
8	Vests	1.17	(0.56)	–0.36	–0.03	0.16	–0.00	0.08	0.15	0.35	–			
9	Lessons/themes	0.79	(0.17)	–0.14	–0.03	–0.01	–0.01	0.13	0.12	0.56	0.19	–		
10	Game	0.35	(0.22)	–0.26	–0.14	0.15	0.03	0.06	0.09	0.25	0.18	0.32	–	
11	Persistent	0.42	(0.49)	0.10	0.23	–0.10	0.09	0.13	0.16	0.16	0.14	0.15	0.06	–

Staff reports on coordination, planning and informing are weakly positively interrelated (0.13–0.19). Planning and informing are also weakly positively related to student perceptions of the program use (i.e. vests, lessons, and game, 0.06–0.15) suggesting that all these variables may reflect an overall level of fidelity in implementation, but the weak level of the correlations indicate the different schools may emphasize different parts of the program. The correlations are small to moderate among the variables measuring implementation of the program as perceived by students (0.18–0.32). Stronger correlations are found between students' awareness of the KiVa program with having had KiVa lessons (0.56), having played the online games (0.25), and the recess supervisors' vests being used (0.35), which is quite natural because these elements are clearly visible to students as well as being explicitly designed to raise student awareness.

**Figure 2:**
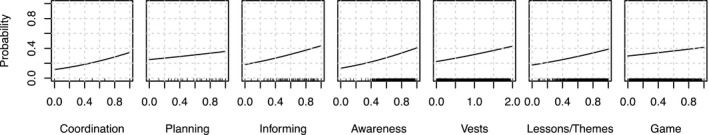
Marginal prediction plots for the likelihood of belonging to the persistent group based on logistic regression results.

The results from the logistic regression analysis including all predictors are shown in Table 5. The negative effect of baseline victimization implies that schools with less victimization to begin with are more likely to persist with the program. School size (larger schools), coordination, informing, student awareness of KiVa, student perceptions of KiVa vests being used, as well as having had KiVa lessons/themes are statistically significantly related to persistent participation. Figure 2 presents the magnitudes of the effects graphically as marginal prediction plots of one variable at a time holding all other variables in the model at their means. The effects of planning and game, although positive, were not statistically significant and thus the evidence from the study does not allow us to conclude the existence of these effects. Lessons/themes, awareness, and informing have the largest effects. For each of these effects, the expected probability of being in the persistent group doubles from less than 20% to more than 40% between the schools that scored the least compared to the schools that scored the most on these variables.

**Table 5:** Logistic regression analyses predicting persistence

	Estimate	(*SE*)	*p*‐value	*OR*	95% CI
Intercept	−6.08	(0.876)	< 0.001		
Middle school	0.26	(0.14)	0.063	1.30	[0.99, 1.71]
School size/100	0.31	(0.04)	< 0.001	1.36	[1.26, 1.46]
Initial victimization	−0.55	(0.26)	0.034	0.58	[0.34, 0.96]
Coordination	1.40	(0.64)	0.028	4.06	[1.16, 14.14]
Planning	0.52	(0.37)	0.152	1.69	[0.83, 3.45]
Informing	1.24	(0.39)	0.002	3.44	[1.60, 7.39]
Awareness	1.49	(0.73)	0.040	4.45	[1.07, 18.47]
Vests	0.48	(0.12)	< 0.001	1.62	[1.27, 2.06]
Lessons/themes	1.10	(0.45)	0.016	2.96	[1.23, 7.13]
Game	0.51	(0.26)	0.081	1.66	[0.94, 2.94]

## Discussion

While there is considerable evidence on the effectiveness of evidence‐based prevention and intervention programs, researchers have raised concerns on the sustainability of these programs (e.g., Bumbarger & Perkins, 2008; Fixsen *et al*., 2017); some go as far as arguing that ignoring the sustainability of the programs after their initial evaluations is an ethical problem (Scheirer & Dearing, 2011). Indeed, programs that have proven to be effective should not remain as short‐term projects, but instead be integrated into schools' everyday practices. In this study, we focused on sustainability of the KiVa antibullying program, which was successfully scaled up in Finland in 2009.

We identified four implementation profiles, which were highly similar to the ones identified by McIntosh, Mercer, Nese, and Ghemraoui (2016). The *persistent* schools (43%) maintained a high likelihood of participation throughout the study period (cf. the *sustainers*, 29% in McIntosh *et al*.). The *awakened* (14%) started lower than average with a decreasing trend during the first years, but then again increasing the likelihood of program participation (cf. the *slow starters*, 13% in McIntosh *et al*.). Furthermore, two groups with a declining trend in participation were identified, the *tail‐offs* (20%) and the *drop‐offs* (23%; cf. the *late abandoners*, 24%, and the *rapid abandoners*, 34%, in McIntosh *et al*.) The likelihood of participation declined either after the first (drop‐offs) or the third year (tail‐offs) of implementation, thus being in line with the suggestion by McIntosh, Mercer, Nese, and Ghemraoui (2016) that these years were especially fragile periods for abandoning the programs. These findings suggest, that many schools need support during the initial years to launch and maintain the implementation of the evidence‐based programs.

We found that school size was related to the sustainability of program participation. More specifically, similar to McIntosh, Mercer, Nese, and Ghemraoui (2016), larger schools were more likely to sustain the program than smaller ones. It is possible that a multicomponent whole‐school program appears too overwhelming for small school. Also, small schools may be more vulnerable for staff turnover, which challenges the maintenance of the practises.

We also found that schools with lower baseline levels of victimization were more likely to sustain the program. A likely explanation for this finding is that the schools that are doing well to begin with have more capacity to adopt a new program. The fit between the program and the organizations' values and practises has, indeed, been suggested as a predictor for sustainability in the qualitative studies (Andreou *et al*., 2015; Leadbeater *et al*., 2015; Woodbridge *et al*., 2014). Possibly, the elements from the program can be adopted more easily when they are not too far from the previous practises; and moreover, the additional workload to be accepted, if it is assumed as a natural part of work instead of being viewed as something extra over the regular work.

Moving on to the main focus, the actionable program elements and implementation practices that are under schools' control, we found that having active purveyors or agents promoting the program at school is important for sustainability. It is crucial to have a staff member or members who are in charge of the program coordination, especially with multicomponent programs with a whole‐school approach to prevent and tackle bullying. Such programs are not necessarily kept alive if no one is speaking for them and reminding about the actions that need to be taken. These findings corroborate the qualitative interviews by Leadbeater *et al*. (2015), where several of their participants brought up the theme of “requiring ongoing communication and renewing of commitments to sustain the program” (p. 126). In accordance, our findings also implied that informing the whole school community of the program predicted sustainability. Thus, besides having the active agents responsible of the program maintenance, engaging the whole school, so that each and every one is at least aware of the program, should be viewed important for sustaining evidence‐based programs.

Finally, we found that the delivery of student lessons, as well as recess supervisors' use of the vests during the initial years of implementation, in other words the concrete tasks that are visible to all members of the school, can be important for long‐term commitment. Therefore, when funding the implementation of evidence‐based programs, it is important to put enough effort in the initial visibility of the program in the schools. The initial commitment is also likely to show in the effects obtained (i.e., reduced bullying), further encouraging the continuation of the program.

### Limitations and future directions

We used the schools' participation in the annual online survey across seven years as a measure of sustainability. The strength of this approach is that it does not rely on retrospective data, or rely purely on the principal or staff points of view, but rather reflect actual activity of the program. However, this measure does not reflect *sustaining the fidelity* or *quality* of implementation (as raised important by Fixsen *et al*., 2017; and Han & Weiss, 2005). As high‐fidelity implementation of bullying prevention practices is related to program outcomes (Haataja *et al*., 2014; Hirschstein, Van Schoiack Edstrom, Frey, Snell & MacKenzie, 2007; Low, Smolkowski & Cook, 2016), future studies should assess the degree and quality of implementation when measuring sustainability. At the same time, attention should be paid to program adaptations and modifications. Some adaptations may be necessary when integrating the program into school practices (Harn *et al*., 2013; Owens *et al*., 2014). However, this should not happen at the cost of reduced effectiveness of the program. Therefore, the identification of the core elements producing the program effects is crucial along with revealing the modifications that are appropriate (Harn *et al*., 2013; Owens *et al*., 2014).

Missing data is an obvious limitation in this kind of longitudinal study in a natural setting without researchers intervening and reminding of responding; it was especially problematic in the case of the staff survey. While modern missing data techniques can compensate for data that is missing at random, it is always possible that the data are not missing at random leading to biased results. In our study, we believe that the effect of missing data in the staff survey is such that missingness correlates with smaller values in the staff variables and also smaller probability to be in the persistent group. As explained in the methods section, this mechanism would lead to underestimation of the effects of coordination, planning, and informing. These estimates may thus be conservative, with real effects being stronger.

Moreover, the design‐based missingness added to challenges as the larger first cohort could not respond to implementation questions added in 2011. In future studies, more attention is needed to setting up the data collection. First, the schools would need to understand the importance of monitoring their implementation along with providing trend data on outcomes. This would help recognizing when implementation goes in a wrong direction, while research would benefit from the data. Second, planning long‐term implementation should start already before the intervention begins so that the implementation would be measured from the very beginning.

Related to the data collection, we asked only a single person from each school to respond the staff survey. This decision could be questioned as possibly giving biased view from the implementation characteristics. Other staff members could view the situation differently, which could also be an important question for future studies.

Considering the predictors, there are naturally several factors not evaluated in this study that can further predict sustainability. For instance, we did not measure principal commitment, which has been found to be connected to delivering more KiVa lessons in the previous studies (Ahtola *et al*., 2013; Haataja *et al*., 2015), and also suggested as an important predictor of sustainability in the qualitative studies (Leadbeater *et al*., 2015). It is also possible that the persistent schools have some pre‐implementation features that explain their success in sustainable implementation. For instance, the antibullying values and practices prior to program implementation could predict sustainability, and likely also explain the low baseline level of victimization. Overall, examining the pre‐existing conditions, or as Fixsen *et al*. (2017) state, organizational capacities to implement a program, would be important in order to deepen the current knowledge on the factors that affect sustainability. Moreover, it would be important to take into account the uncontrollable, time‐varying factors such as staff turnover.

Finally, as Fixsen *et al*. (2017) noted, more attention should be paid to de‐adoption and re‐adoption of the program. Given the different critical years for abandoning the program, future studies should focus on specific factors related to drop‐offs and tail‐offs. It is relevant to understand the reasons behind abandoning the program after the first year (i.e., drop‐offs) and after a couple of years (i.e., tail‐offs) in order to provide tailored support for the schools in the beginning of implementation and after having worked with a program for some years.

### Practical implications

The findings have important implications for bringing evidence‐based antibullying programs into real‐life school contexts. First, although a large proportion of schools managed to sustain the KiVa program for several years, nearly half of the schools seemed to abandon it in the very beginning or after a couple of years. It is important that resources are not used only to evaluate and launch evidence‐based programs, but also to support schools in these critical time points. Second, initial training and support should guide schools in planning sustainability. For instance, schools should from the very beginning assign person(s) in charge to coordinate and maintain the program. Moreover, informing the whole school community about a prevention program is important for the process of integrating the program in the schools' everyday life.

## Notes

1 In spring 2016, 92% of Finnish schools providing comprehensive education were registered KiVa program users (*n* = 2,268). Total number of schools in Finland in 2016 was 2,455 (Official Statistics of Finland, OSF, 2016).

2 We considered also using the three‐step process presented by Asparouhov and Muthen (2014) in a mixture model where the estimated misclassification rates from the LCA analysis are used to define some of the parameters in the estimated categorical outcome model. However, FIML estimation was not available for this kind of model necessitating the use of multiple imputation instead, and thus needlessly complicating the analysis.
